# The Impact of Ecological Civilization Theory on University Students’ Pro-environmental Behavior: An Application of Knowledge-Attitude-Practice Theoretical Model

**DOI:** 10.3389/fpsyg.2021.681409

**Published:** 2021-12-01

**Authors:** Kuan Wang, Lu Zhang

**Affiliations:** ^1^School of Marxism, Liaoning University, Shenyang, China; ^2^College of Aerospace Engineering, Shenyang Aerospace University, Shenyang, China

**Keywords:** ecological civilization theory, environmental knowledge, pro-environment behavior, KAP theoretical model, environmental education in China

## Abstract

In environmental education, environmental knowledge is considered to be one of the most important factors affecting university students’ pro-environmental behavior. First, in this paper, the ecological civilization theory (ECT) was understood as a new kind of environmental knowledge. Based on this, a new theoretical model for analyzing the relationships among environmental knowledge, environmental attitude, and environmental behavior was designed in this paper according to ECT and the Knowledge-Attitude-Practice (KAP) theoretical model. Second, from the perspective of students, a questionnaire was designed for students according to ECT, so as to understand the level of ECT of students. On this basis, an empirical test of the relationship between the ECT level, pro-environmental attitude level, and pro-environmental behavior level was carried out. This research shows that ECT as environmental knowledge is as important as science-oriented environmental knowledge (SEK) in environmental education. As a result, the role of environmental knowledge in environmental education should not be ignored but environmental knowledge should be enriched by adding ECT to the environmental knowledge system and improving the environmental knowledge education curriculum, contributing to environmental education in China.

## Introduction

In recent years, not much has improved in the Chinese ecological environment, which has attracted the attention of the Chinese government to formulate systematic environmental protection strategies and policies, design environmental education programs, and encourage university students’ pro-environmental behavior (Wang et al., 2021).

It is widely agreed that current human behavior has a negative impact on the natural environment (Edgell and Nowell, 1989; IPCC, 2014). Thus, avenues for increasing pro-environmental behavior are required. Environmental education can serve as a critical tool in increasing pro-environmental behavior as it strives toward the goal of environmental protection (Potter, 2009; Ariffin and Wan, 2017). Environmental education is the comprehensive education of environmental knowledge, environmental attitude, and ecological behavior. Environmental education aims to motivate people to perform appropriate real-life pro-environmental behavior (Carmi et al., 2015; Roczen et al., 2015). Indeed, environmental education is regarded as an indispensable requirement if we want to increase pro-environmental behavior and protect the natural environment successfully (Fortner and Teates, 1980; Michelsen and Fischer, 2017).

In environmental education, environmental knowledge is important in producing pro-environmental behavior because an individual must know what type of actions needs to be taken. Thus, environmental knowledge is an intellectual prerequisite to performing pro-environmental behavior (Stern and Gardner, 2002; Frick et al., 2004; Fujii, 2006; Kaiser et al., 2014; Nordin and Saliluddin, 2016). People who have greater knowledge of environmental problems are more prone to behave in a pro-environmental way, ceteris paribus (Oguz et al., 2010). On the contrary, a shortage of environmental knowledge or the holding of wrong environmental perception might limit pro-environmental behavior. A study in Hungary found that more than 50% of the respondents felt that their pro-environmental behavior was often constrained by a shortage of environmental knowledge (Zsóka et al., 2012).

The reality, though, is more complicated than it seems. The relation between environmental knowledge and pro-environmental behavior has been disputed (Frick et al., 2004; Geiger et al., 2014; Gharagozlou et al., 2019), and maybe influenced by several factors, such as motivational components in the form of personal attitude (Gatersleben et al., 2002; Gifford and Nilsson, 2014). Previous research investigating the relationship between environmental knowledge and pro-environmental behavior shows that environmental knowledge, more often than not, fails to directly influence pro-environmental behavior (Hines et al., 1986–1987; Kals et al., 1999; Kaplowitz and Levine, 2005; Steg and Vlek, 2009; Levy and Marans, 2012; Kastanakis and Voyer, 2014). In other words, in environmental education, environmental knowledge and environmental attitude work together to produce pro-environmental behavior. As a result, a more complex theoretical model of environmental education has been produced. Knowledge-Attitude-Practice (KAP) is a behavioral intervention theory, which is one of the common models to explain how environmental knowledge affects pro-environmental behavior (Milfont, 2009; Paço and Lavrador, 2017). It was first proposed by the British scientist John Coster in the 1960s.

At present, environmental education in Chinese universities still pays attention to the influence of environmental knowledge on environmental behavior, and mainly focuses on the teaching of environmental knowledge. Environmental knowledge can be defined as a general knowledge of facts, concepts, and relationships concerning the natural environment and its major ecosystems (Fryxell and Lo, 2003; Liobikienė et al., 2016). Environmental knowledge involves what people know about the environment, key relationships leading to environmental aspects or impacts, an appreciation of “whole systems,” and collective responsibilities necessary for sustainable development (Kaiser et al., 2014). Environmental knowledge is usually divided into three categories: system knowledge, action knowledge, and efficiency knowledge (McGorry, 2000; Geiger et al., 2018).

However, to date, a lot of research studies on environmental knowledge have examined only one or, at most, three forms of environmental knowledge. In the course of daily environmental education, an interesting phenomenon was found in this paper. Students who took some courses that included the ecological civilization theory (ECT) of the Chinese government showed more pro-environmental behavior. These courses are mainly ideological and political theory courses. Then, will the ECT of the Chinese government become a new type of knowledge that affects pro-environmental behavior? As a result, a new theoretical model for analyzing the relationships among environmental knowledge, environmental attitude, and environmental behavior were designed in this study based on ECT and the KAP theoretical model, contributing to environmental education in China.

## Literature Review

From the current situation of environmental education in Chinese universities, after nearly 20 years of difficult exploration, some achievements have been made, but there are still some shortcomings, mainly manifested in the insufficient education of ECT. There are not many theoretical works on it in China, and the understanding in practice is still insufficient. It is necessary to conduct a further study on it to realize its guidance and promotion in practice.

The ultimate goal of environmental education is to achieve the development of pro-environmental behavior and the formation of real problem-solving capabilities (Mostafa, 2007; Ottoa and Pensinib, 2017). Therefore, environmental education as an effective way to implement the development of students’ core qualities will inevitably become the trend of curriculum reform and development. For environmental education, a large number of worldwide scholars have conducted research. Their main research focus is to explore the potential factors that influence students’ pro-environmental behavior, so as to actively explore such potential factors and help students improve their pro-environmental behavior. Environmental knowledge and attitude are considered to be the most important factors affecting pro-environmental behavior. Therefore, the main content of environmental education is environmental knowledge teaching. Through an increase of environmental knowledge, pro-environmental attitude is improved, and then pro-environmental behavior is improved. Zsóka et al. (2012) explore the relationship between environmental education and environmental knowledge, attitude, and the reported actual behavior of university and high school students. Kaiser et al. (2014) begin by distinguishing the three forms of environmental knowledge and go on to predict that people’s attitude toward nature represents the force that drives their pro-environment behavioral engagement. Based on the data from 1,907 students, Kaiser et al. (2014) calibrated the previously established instruments to measure pro-environmental behavior, environmental knowledge, and attitude toward nature with Rasch-type models. Vicente-Molina et al. (2013) analyze the influence of environmental knowledge on pro-environmental behavior among university students from countries with different levels of economic development. The results suggest that motivation and perceived effectiveness are not only significant variables in both groups but also the most important ones in explaining pro-environmental behavior. Genovaite and Mykolas (2019) have transformed Value-Belief-Norm (VBN) by including environmental knowledge as an external factor. The results showed that action-related environmental knowledge was related to an ecological worldview and directly influenced the private sphere behavior. Thus, Genovaite and Mykolas (2019) revealed how specific environmental knowledge influenced pro-environmental behavior.

Based on the current study, new research was designed in this paper. In this study, ECT was understood as a new kind of environmental knowledge. From the perspective of students, a questionnaire was designed for students according to ECT to understand students’ ECT level once again. On this basis, an empirical test of the relationship between the ECT level, pro-environmental attitude level, and pro-environment behavior level was carried out, and then the validity of the KAP theory in environmental education was proved. Based on the conclusion of this study, we will evaluate the existing environmental education in China and put forward suggestions for improvement.

## Materials and Methods

### Knowledge-Attitude-Practice in Environmental Education

The theory divides the change in human practice into three continuous processes: acquiring knowledge, producing attitude, and forming practice (Prothero, 1990). Among them, “knowledge” is an understanding of relevant information, “attitude” is correct belief and positive attitude, and “practice” is behavior. Knowledge produces attitude, and attitude is the result of knowledge. University students’ environmental knowledge can foster positive environmental attitude (Otto and Kaiser, 2014; Tam and Chan, 2018). Environmental attitude changes positively as environmental knowledge increases. People with more knowledge of natural resources have more positive attitude toward environmental protection (Cappetta and Magni, 2015). For example, aquarium visits and publications related to marine protected areas can greatly improve the marine environmental protection attitude of visitors by increasing their knowledge (Ting and Cheng, 2017). Behavior is the external expression of attitude, and attitude has a positive influence on behavior (Taufique et al., 2016; Sarkis, 2017). Environmental attitude is essential to environmental behavior research, and environmental education often seeks ways to determine and modify environmental attitude about the relationship between humans and nature. The main focus has been that, by understanding attitude, environmental education research can better predict the student’s behavior, thereby changing students’ attitude to elicit appropriate environmental behavior (Barber and Taylor, 2009; Yadav and Pathak, 2016).

**FIGURE 1:**
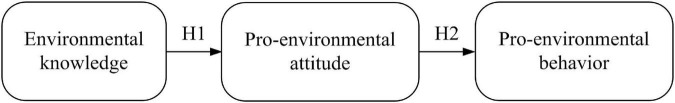
Classical Knowledge-Attitude-Practice (KAP) theoretical model.

Based on this, the classical KAP theoretical model forms two hypotheses with inherent relevance, and the two hypotheses are proved to be valid through empirical methods. Hypothesis 1 (H1): Environmental knowledge positively influences pro-environmental attitude. Hypothesis 2 (H2): Pro-environmental attitude positively influences pro-environmental behavior. Therefore, the classical KAP theoretical model supports environmental education with environmental knowledge as the main teaching content being carried out in China. Knowledge is the premise and foundation for a person to form a psychological tendency of liking or disliking something. Environmental knowledge can promote a pro-environmental attitude. For example, consumers with environmental knowledge are more likely to understand the significance and value of buying green products. Attitude is a person’s evaluation of an object. It expresses a psychological tendency to like or dislike something, or a specific emotional tendency toward something (Paço and Lavrador, 2017), as a significant predictor for interpreting and promoting behavioral intentions. A pro-environmental attitude can promote pro-environmental behavior (Fishbein and Ajzen, 2010). For example, consumers with a positive attitude are more likely to buy “green” and energy-efficient products, they do not find it inconvenient to buy green products. According to the representation of environmental education in the classical KAP model, the relationship among environmental knowledge, pro-environmental attitude, and pro-environmental behavior is shown in Figure 1.

In addition to the classical KAP theoretical model, some scholars proposed an improved theory of the KAP theoretical model (Pisano and Lubell, 2017; Geiger et al., 2018). In the improved KAP theoretical model, the influence of environmental knowledge on pro-environmental behavior is strengthened. The transfer of environmental knowledge to enable people to reflect on their actions rationally and then to act intentionally based on this has been a dominant model of environmental education. At the same time, in the improved KAP theoretical model, a direct influence of environmental knowledge on pro-environmental behavior is also further revealed and verified by empirical research. In the improved KAP theoretical model, environmental knowledge [science-oriented environmental knowledge (SEK)] is divided into three types: system knowledge, action knowledge, and efficiency knowledge (Razak et al., 2015; Geiger et al., 2018). System knowledge refers to the knowledge about the basic operating state and internal laws of the natural ecosystem. For example, global warming is due to the emission of CO_2_, less forest will lead to soil erosion, and shrinking lakes will lead to climate drought. Action knowledge refers to the knowledge about what actions are best for environmental protection. For example, using recyclable bags helps protect the environment, proper disposal of waste batteries helps protect the environment, and rejecting the use of wildlife products helps protect biodiversity. Efficiency knowledge refers to the knowledge about saving energy and saving resources. For example, waste recovery can effectively save resources, taking public transport is conducive to energy conservation, and using energy-saving bulbs can save energy.

**FIGURE 2:**
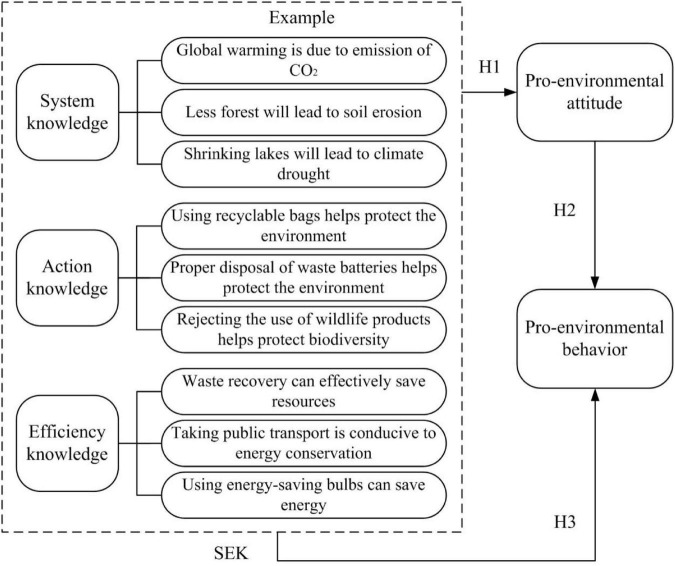
Improved KAP theoretical model.

Based on this, the improved KAP theoretical model forms three hypotheses. Hypothesis 1 (H1): SEK positively influences pro-environmental attitude. Hypothesis 2 (H2): Pro-environmental attitude positively influences pro-environmental behavior. Hypothesis 3 (H3): SEK positively influences pro-environmental behavior. According to the representation of environmental education in the improved KAP theoretical model, the relationship among environmental knowledge, pro-environmental attitude, and pro-environmental behavior is shown in Figure 2.

Based on the abovementioned research, a new KAP theoretical model is studied in this paper. According to the representation of environmental education in the new KAP theoretical model, the types of environmental knowledge are further increased. Because the environmental education experience from China shows that a politics-oriented ECT is an important factor affecting students’ pro-environmental behavior. In the new KAP theoretical model, ECT, as a new type of environmental knowledge, is regarded as an equally important variable as SEK.

**FIGURE 3:**
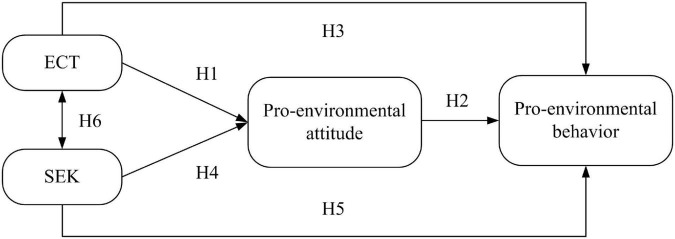
New KAP theoretical model.

Based on this, the new KAP theoretical model forms six hypotheses. Hypothesis 1 (H1): ECT positively influences pro-environmental attitude. Hypothesis 2 (H2): Pro-environmental attitude positively influences pro-environmental behavior. Hypothesis 3 (H3): ECT positively influences pro-environmental behavior. Hypothesis 4 (H4): SEK positively influences pro-environmental attitude. Hypothesis 5 (H5): SEK positively influences pro-environmental behavior. Hypothesis 6 (H6): SEK and ECT interact. Given the abovementioned hypotheses, a research framework is developed for this study as shown in Figure 3.

### Ecological Civilization Theory in Environmental Education

”Ecological prosperity leads to civilization, while ecological decline leads to civilization decline” reflects the importance China attaches to ecological civilization construction. Ecological civilization is a major issue related to the sustainable development of China. Therefore, the theory of ecological civilization is regarded as environmental knowledge that Chinese university students must learn and is an important part of environmental education in China.

To implement the ecological environment protection strategy, China’s environmental education was divided into two groups: science- and politics-oriented environmental education (Wang et al., 2021). Science-oriented environmental education has been continuing since the 1990s and mainly includes elementary, middle, and high school basic education on environmental pollution, ecosystem function, saving resources, and other basic environmental topics. The main teaching contents are basically consistent with the environmental knowledge system in the classical KAP theoretical model. Politics-oriented environmental education has been intensified since 2012 through vigorously promoted strategies, such as ecological civilization, “beautiful China,” and “lucid waters and lush mountains are invaluable assets” *via* various media channels. The main teaching content is an environmental knowledge system different from the environmental knowledge system in the classical KAP theoretical model. To be precise, this environmental knowledge system is ECT.

The ecological civilization theory, as a new type of environmental knowledge, covers political, economic, cultural, social, environmental, and other aspects, mainly including China’s environmental governance strategies, systems, and programs. ECT includes three basic contents: the basic standpoint, the basic viewpoint, and the basic method.

The basic standpoint of ECT is “the people-centered philosophy of development,” which clearly points out that the fundamental purpose of the construction of ecological civilization is not to maintain the intrinsic value of abstract nature, nor to realize sustainable development of economy and society, but to “meet the ecological needs of the people” and realize the ecological happiness of the people. The basic standpoint of ECT reflects a kind of value-related environmental knowledge.

The basic viewpoints of ECT can be summarized as the following six basic viewpoints: the ecological view of “harmonious coexistence of human and nature”; the ecological economic view of “lucid waters and lush mountains are invaluable assets”; the ecological ethics view of “man and nature are life community”; the ecological system view of “accelerating the reform and innovation of the system and mechanism of ecological civilization”; the ecological culture view of “bringing ecological culture into the mass spiritual civilization creation activities”; and the international ecological view of “building an international ecological governance cooperation mechanism featuring extensive consultation, joint contribution, and shared benefits.” The basic viewpoints of ECT reflect a kind of significance-related environmental knowledge.

**FIGURE 4:**
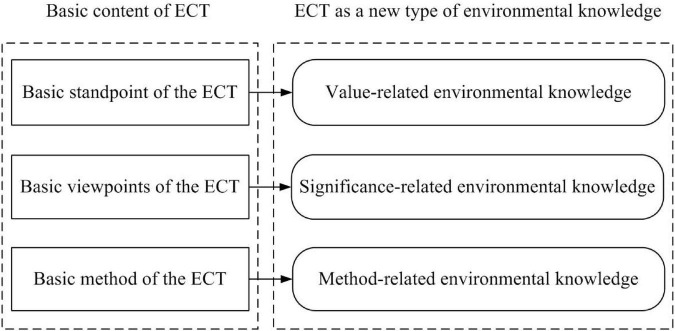
Relationship between ecological civilization theory (ECT) and a new type of environmental knowledge.

The basic method of ECT is the means of China to analyze and deal with China’s environmental problems, including historical analysis, contradiction analysis, and system analysis. According to the historical analysis, the construction of ecological civilization in China is a long process, which needs to be handed down from generation to generation and gradually realizes a harmonious coexistence between man and nature. According to the analysis of contradiction, the main task of ecological civilization construction in China is to solve the contradiction between economic development and environmental protection, which is to realize social development and protect the natural environment as well. According to the system analysis method, China’s ecological civilization construction is a systematic project, which requires us to start from China’s reality, consider environmental governance as a whole, and deal with the wholeness, complexity, and coordination of ecological civilization construction. The basic method of ECT reflects a kind of method-related environmental knowledge. The relationship between the basic content of ECT and ECT as a new type of environmental knowledge is shown in Figure 4.

At present, in China’s environmental education system, there is no specialized course on the theory of ecological civilization, and in environmental education, the theory of ecological civilization as environmental knowledge has not been paid enough attention. The theory of ecological civilization is being taught to university students as part of the ideological and political theory course. However, students who have taken ideological and political theory courses show stronger pro-environmental behavior, which will provide important implications for environmental education in China. A questionnaire about university students’ level of ECT is designed in this study, which is the basis of all the research studies in this paper.

### Data Collection

#### Participants

The data in the valid questionnaires were analyzed and processed to obtain the situation of students’ environmental education. Because the environmental education courses in different universities in China are not completely consistent. Therefore, the participants of this study come from the two universities in the Liaoning Province, China, who receive different types of environmental education courses and meet the special requirements of this study. Some of the students (*n* = 105) had only taken environmental education courses related to ECT. Some of the students (*n* = 107) had only taken environmental education courses related to SEK. Some of the students (*n* = 95) had taken both. Some of the students (*n* = 114) were yet to take any environmental education courses. Among the 421 questionnaires that were completed and returned, 16 were invalid and thus excluded based on the data verification requirements, hence 96.1% of the returned questionnaires (405 questionnaires) were valid and used for research.

#### General Environmental Behavior Scale

General environmental behavior was measured by a comprehensively tested and validated eight-item self-report instrument (Midden et al., 2007). The instrument originally had 40 items, but it was reduced to eight items for this study. For eight of the environmental behavioral self-report items, we used a 5-point scale ranging from 1 (never) to 5 (always).

#### General Environmental Attitude Scale

The general environmental attitude was measured by a comprehensively tested and validated eight-item self-report instrument (Brügger et al., 2011). The instrument originally had 40 items, but it was reduced to eight items for this study. For eight of the environmental attitude self-report items, we used a 5-point scale ranging from 1 (totally disagree) to 5 (in full agreement).

#### General Environmental Knowledge Scale

General environmental knowledge (SEK) was measured by a comprehensively tested and validated eight-item self-report instrument (Frick et al., 2004). The instrument originally had 48 items, but it was reduced to eight items for this study. For eight of the environmental knowledge self-report items, we used a 5-point scale ranging from 1 (no knowledge) to 5 (biggest knowledge).

#### Ecological Civilization Theory Scale

The level of ECT was measured by a comprehensively tested and validated 20-item self-report instrument (Wang et al., 2020). The 20 questions are newly developed for this particular research. According to the opinions of experts in environmental education and ideological and political courses, we design a set of questionnaires reflecting ECT. The questionnaire is designed according to the basic content of ECT. It reflects the value-related environmental knowledge of ECT, the significance-related environmental knowledge of ECT, and the method-related environmental knowledge of ECT. The items of the questionnaire were emailed to eight relevant experts of China who studied environmental education and ideological and political courses, and the experts were asked to score the items of a questionnaire to ensure the accuracy of the measurement. Finally, the experts gave scores based on the years of experience and relevant public research questionnaires. All 20 items were measured by using a 5-point scale ranging from 1 (totally disagree) to 5 (in full agreement). Experts give scores (*M* = 4.750, *SD* = 0.463) well above 4, which indicate that the experts agree with the evaluation system of this paper, and the ECT evaluation method is feasible and the opinions of experts are almost unified. For 20 of the ECT self-report items, we used a 5-point scale ranging from 1 (not at all) to 5 (very clear). The ECT scale is shown in Table 1.

**TABLE 1:** Ecological civilization theory (ECT) scale.

Value-related environmental knowledge	Not at all	Not really	General	Know better	Very clear
(1) Do you know the ecological happiness must be taken as the core of environmental governance?	1	2	3	4	5
(2) Do you know the fundamental goal of China’s ecological progress is to realize the ecological happiness of the people?	1	2	3	4	5
(3) Do you know the construction of ecological civilization in China requires the joint participation of all citizens?	1	2	3	4	5
(4) Do you know all people should share the fruits of China’s ecological progress?	1	2	3	4	5
(5) Do you know the happiness is primary purpose of ecological civilization construction?	1	2	3	4	5
(6) Do you know the basic standpoint of the ecological civilization theory is the “the people-centered philosophy of development”?	1	2	3	4	5

**Signification-related environmental knowledge**	**Not at all**	**Not really**	**General**	**Know better**	**Very clear**

(7) Do you know the direction and focus of China’s ecological progress is to give priority to prevention and give priority to source control in environmental protection?	1	2	3	4	5
(8) Do you know the core of ecological civilization is the harmonious coexistence between man and nature?	1	2	3	4	5
(9) Do you know the ecological view of “harmonious coexistence of human and nature”?	1	2	3	4	5
(10) Do you know the ecological economic view of “lucid waters and lush mountains are invaluable assets”?	1	2	3	4	5
(11) Do you know the ecological ethics view of “man and nature is life community”?	1	2	3	4	5
(12) Do you know the ecological system view of accelerating the reform and innovation of the system and mechanism of ecological civilization?	1	2	3	4	5
(13) Do you know the international ecological concept of building an international ecological governance cooperation mechanism featuring extensive consultation, joint contribution and shared benefits?	1	2	3	4	5

**Method-related environmental knowledge**	**Not at all**	**Not really**	**General**	**Know better**	**Very clear**

(14) Do you know the construction of ecological civilization in China is a long process?	1	2	3	4	5
(15) Do you know the basic principle of harmonious coexistence between man and nature is to conform to nature?	1	2	3	4	5
(16) Do you know the main task of ecological civilization construction in China is to resolve the conflict between economic development and environmental protection?	1	2	3	4	5
(17) Do you know the methodology of Chinese ecological civilization is historical analysis, System analysis, Contradiction analysis?	1	2	3	4	5
(18) Do you know the basic method of building resource-conserving and environment-friendly society is sustainable development?	1	2	3	4	5
(19) Do you know the systematic analysis method of ecological civilization construction in China is the overall analysis, structure analysis and collaborative analysis?	1	2	3	4	5
(20) Do you know the ecological civilization construction in China is a systematic project?	1	2	3	4	5

First, we divided the students into four groups. Group A (*n* = 105) only received ECT education. Group B (*n* = 107) only received SEK education. Group C (*n* = 95) received SEK education and ECT education. Group D (*n* = 114) received no environmental education. Second, the general environmental knowledge scale and ECT scale were, respectively, used to test the four groups of university students to examine their environmental knowledge level. Next, the general environmental behavior scale and general environmental attitude scale were, respectively, used to test the four groups of university students to examine their pro-environmental behavior level and pro-environmental attitude level. Finally, the correlation coefficient between SEK, ECT, pro-environmental attitude, and pro-environmental behavior of the four groups of students was calculated to prove the important role of ECT in environmental education.

## Results

We will present the details of our confirmatory test of the theoretically anticipated relations between environmental knowledge (SEK and ECT), pro-environmental attitude, and pro-environmental behavior.

### Descriptive Analysis of the Obtained Data

**FIGURE 5:**
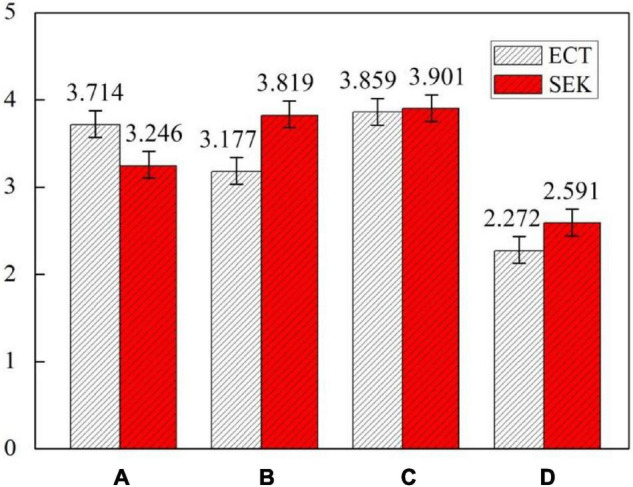
Level of science-oriented environmental knowledge (SEK) and ECT of groups A, B, C, and D.

Environmental knowledge (SEK and ECT) level of groups A, B, and C who have attended environmental education courses (*M* = 3.619, *SD* = 1.035) is much higher than group D (*M* = 2.431, *SD* = 1.612) who have not received environmental education. The environmental knowledge (SEK and ECT) level of the overall four groups (*M* = 3.322, *SD* = 1.426) is higher than 3, which is a high level. This shows that China’s environmental education has achieved some achievements, but it has not reached a satisfactory level. The ECT level of group A (*M* = 3.714, *SD* = 0.904) and group C (*M* = 3.859, *SD* = 0.897) who have attended related courses of ideological and political theory are higher than the overall four groups (*M* = 3.255, *SD* = 1.276). This shows that China’s ideological and political theory courses are effective in ECT education. The SEK level of group B (*M* = 3.819, *SD* = 0.885) and group C (*M* = 3.901, *SD* = 0.859) who have attended related courses of the SEK are higher than the overall four groups (*M* = 3.389, *SD* = 1.290). This shows that China’s environmental education courses are effective in SEK education. Students in group C (*M* = 3.880, *SD* = 1.103) who have taken two environmental knowledge courses at the same time had the highest environmental knowledge (SEK and ECT) level, while students in group D (*M* = 2.431, *SD* = 1.612) who have not taken any environmental education courses had the lowest environmental knowledge (SEK and ECT) level. The level of environmental knowledge (SEK and ECT) of groups A, B, C, and D are shown in Figure 5.

The pro-environmental attitude level of groups A, B, and C who have attended environmental education courses (*M* = 3.548, *SD* = 1.114) is much higher than group D (*M* = 2.579, *SD* = 1.512) who have not received environmental education. The pro-environmental attitude level of group C (*M* = 3.814, *SD* = 0.859) who have taken two environmental knowledge courses at the same time had the highest level of pro-environmental attitude among all the four groups. While students in group D (*M* = 2.579, *SD* = 1.412) who have not taken any environmental education courses had the lowest pro-environmental attitude level. The pro-environmental attitude level of the overall four groups (*M* = 3.305, *SD* = 1.226) is higher than 3, which is a high level. This shows that Chinese university students generally have high pro-environmental attitude, which again proves that China’s environmental education has achieved certain achievements.

**FIGURE 6:**
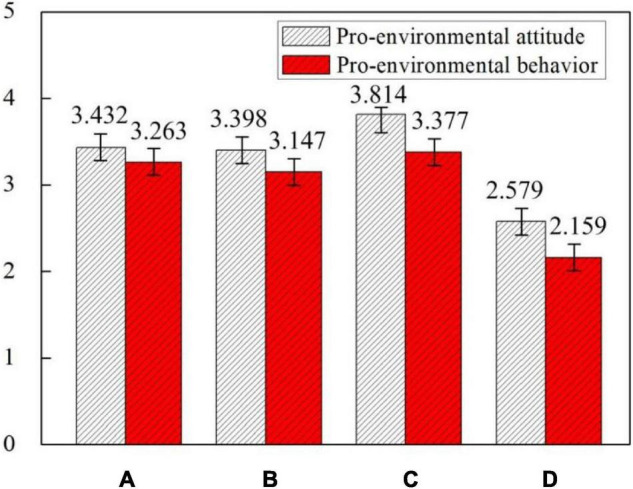
Levels of pro-environmental attitude and pro-environmental behavior of groups A, B, C, and D.

The pro-environmental behavior level of groups A, B, and C who have attended environmental education courses (*M* = 3.262, *SD* = 1.119) is still much higher than group D (*M* = 2.159, *SD* = 1.865) who has not received environmental education. The pro-environmental behavior level of group C (*M* = 3.377, *SD* = 0.797) is still the highest among the four groups. However, there are no significant difference between group C (*M* = 3.377, *SD* = 0.797), group A (*M* = 3.263, *SD* = 0.959), and group B (*M* = 3.147, *SD* = 1.231). The pro-environmental behavior level of the overall four groups (*M* = 2.986, *SD* = 1.445) is almost equal to 3, which is still a good level. The levels of pro-environmental attitude and pro-environmental behavior of groups A, B, C, and D are shown in Figure 6.

### Correlation Analysis Between Ecological Civilization Theory, Science-Oriented Environmental Knowledge, Pro-environmental Attitude, and Pro-environmental Behavior

The correlation coefficient between ECT and pro-environmental attitude is 0.571 (*p* < 0.05), which is a high correlation degree. This supported hypothesis H1. This shows that ECT does have a significant impact on pro-environmental attitude, and the more ECT, the stronger pro-environmental attitude will be. The correlation coefficient between pro-environmental attitude and pro-environmental behavior is 0.214 (*p* < 0.05), which is a weak correlation. However, this still supported hypothesis H2. This shows that the ECT level of university students does not necessarily translate into 100% pro-environmental behavior through pro-environmental attitude, and pro-environmental behavior is also influenced by objective factors such as economic factors, which is also confirmed in the interviews with students. This has created an opportunity for the perfection of the KAP theoretical model in environmental education. The correlation coefficient between SEK and pro-environmental attitude is 0.561 (*p* < 0.05), indicating a high correlation degree. This supported the hypothesis H4. This once again proves the effectiveness of the KAP theoretical model in environmental education. The correlation coefficient (*r* = 0.571) between ECT and pro-environmental attitude is almost equal to the correlation coefficient (*r* = 0.561) between SEK and pro-environmental attitude. This shows that ECT, like SEK, had a positive impact on pro-environmental attitude. ECT as environmental knowledge is as important as SEK in environmental education.

The correlation coefficient between ECT and pro-environmental behavior is 0.447 (*p* < 0.05), which is still relatively high. This supported hypothesis H3. This shows that ECT does have a direct impact on pro-environmental behavior. The more ECT, the more frequent pro-environmental behavior is. The correlation coefficient between SEK and pro-environmental behavior is 0.344 (*p* < 0.05), which is a medium correlation. This supported hypothesis H5. The correlation coefficient (*r* = 0.447) between ECT and pro-environmental behavior is greater than that (*r* = 0.344) between SEK and pro-environmental behavior. This shows that SEK produces pro-environmental behavior less quickly than ECT does. This is probably because of the spread of ECT that has been strongly supported by the Chinese government, and students have learned ECT quickly through various channels.

It is worth mentioning that the correlation coefficient between SEK and ECT is 0.618 (*p* < 0.05), which is a high correlation degree, which means that they interact with each other, thereby showing the fusion of the knowledge groups. This supported hypothesis H6. The significant correlation among ECT, SEK, pro-environmental attitude, and pro-environmental behavior is shown in Table 2.

**TABLE 2:** Correlation among ECT, science-oriented environmental knowledge (SEK), pro-environmental attitude, and pro-environmental behavior.

	ECT	SEK	Pro-environmental attitude
Pro-environmental attitude	0.571	0.561	–
Pro-environmental behavior	0.447	0.344	0.214
SEK	0.618	–	0.561

*p < 0.05.*

### Case Analysis of Students

In this paper, 10 students from group C, who received SEK education and ECT education were selected for interview. The interview results are shown as follows.

Through interviews with students, it was found that the students with more environmental knowledge (SEK and ECT) were marked with stronger pro-environmental attitude and pro-environmental behavior. Students who were interviewed pointed out, that compared with SEK, the theory of ecological civilization can better enable students to understand the value, significance, and the method of environmental governance so that students can clearly understand the significance of environmental protection to national development and people’s happiness. This is very useful for improving their pro-environmental attitude and pro-environmental behavior. Therefore, as a kind of environmental knowledge, ECT plays a very important role in environmental education. At the same time, they actively link SEK with the theory of ecological civilization to form a new environmental knowledge system. This is much greater than pro-environmental attitude and pro-environmental behavior that come from just having a kind of environmental knowledge (SEK or ECT). They also suggest that the current courses on the theory of ecological civilization are not very attractive, and students often acquire relevant knowledge from outside the courses. They have obtained a considerable amount of information through various information channels such as the news media. The students also said that, although they have developed pro-environmental attitude through learning environmental knowledge (SEK and ECT), it does not mean that they adopt pro-environmental behavior every time because pro-environmental behavior is also influenced by some objective conditions, such as economic conditions and technological conditions.

On the whole, students pay close attention to the significance conveyed by the theory of ecological civilization. It is because students feel the great significance of ecological civilization through learning that they are more willing to take pro-environmental behavior. At the same time, students also think that the relevant courses are not attractive.

## Discussion

The core of modern environmental education has changed from allowing students to master environmental knowledge to adapting them to life-long pro-environmental behavior (Bhattacharya, 2019). Thus, this paper studies and analyzes the relationship between environmental knowledge, environmental attitude, and environmental behavior of university students, and puts forward that the theory of ecological civilization can be studied as a kind of environmental knowledge that can affect pro-environmental attitude and pro-environmental behavior. In our research, we confirmatory tested the anticipated pro-environmental behavior structure that was originally proposed in the KAP theory model. Specifically, we found that there is a positive correlation between environmental knowledge (SEK and ECT) and environmental attitude, and the influence of pro-environmental attitude on pro-environmental behavior is also significant. In the structure of environmental knowledge, the influence of ECT on pro-environmental attitude is significant, which can lead to pro-environmental behavior, which again proves the hypothesis of this study. However, the conclusion of this study is in conflict with the conclusion of some other studies. For example, some studies that examined systems or knowledge of environmental issues found an insignificant relationship between knowledge and pro-environmental behavior (Rhead et al., 2018). Some studies suggest that environmental knowledge does not necessarily result in pro-environmental actions (Ahmad et al., 2018).

From our results, environmental knowledge (SEK and ECT) does have a direct impact on pro-environmental behavior. However, the correlation between environmental knowledge (SEK and ECT) and pro-environmental behavior is indeed weaker than that between environmental knowledge (SEK and ECT) and pro-environmental attitude. There is, however, a possible explanation for this finding. Because our students knew so little about environmental issues, how systems work, behavioral remedies, especially the significance of ecological civilization construction for national development, range restrictions due to floor effects seemed to occur. In other words, we found the seemingly small knowledge effects that might have been due to our students who extremely restricted the level of environmental knowledge because the restricted variances of variables often lead to artificially deflated correlations with other variables as well (Tabachnick and Fidell, 2006). From our results, we should not conclude that environmental knowledge can be abandoned. A general increase in environmental knowledge, especially an increase of ECT, might in fact already be able to alleviate the weak relations between knowledge and behavior (Kaiser and Frick, 2002).

Thus, the important thing is not to abandon environmental knowledge but to strengthen environmental knowledge. It is important to consider the type of environmental knowledge as well. Two suggestions can be made for China’s environmental education. First, it is suggested that the curriculum system of environmental education should be further improved to improve the environmental knowledge level of university students. More importantly, all students are required to receive environmental education, and SEK and ECT must be taken into account at the same time to achieve the integration of different types of environmental knowledge, so as to achieve a better environmental education effect. In addition, efforts should be made to make the courses of environmental education more vivid and interesting, which should be close to life and arouse students’ interest in learning. Second, it is suggested that the relevant education of ECT is strengthened so that university students can fully understand the significance of ecological civilization construction and its importance for national development. Value-related environmental knowledge, signification-related environmental knowledge, and method-related environmental knowledge provided by ECT, functioning as a heuristic, could reduce the cognitive load needed to make decisions, thus potentially having a direct effect on behavior. There are a few studies showing that people who know about behavior significance and value are more confident and inclined to behave accordingly (Cappetta and Magni, 2015). Thus, there is evidence that significance-related knowledge and value-related environmental knowledge enabling individuals to make concrete and informed decisions might more easily be translatable into behavior.

## Conclusion

In our research, we have demonstrated that both the classical KAP theoretical model and the new theoretical model proposed in this paper are effective in environmental education in China. China’s environmental education has made some achievements, but it is still far from the ideal level. Both ECT and SEK have a significant impact on pro-environmental attitude, and the more ECT and SEK, the stronger pro-environmental attitude will be. Similarly, both ECT and SEK have a direct impact on pro-environmental behavior. The more ECT and SEK, the more frequent pro-environmental behavior is. This shows that ECT as environmental knowledge is as important as SEK in environmental education. However, we also found that the ECT level of university students does not necessarily translate into pro-environmental behavior 100% through pro-environmental attitude, and pro-environmental behavior is also influenced by objective factors such as economic factors, which is also confirmed in the interviews with students. At the same time, we found that SEK in the classical KAP theoretical model produces pro-environmental behavior less quickly than ECT does. This is probably due to the spread of ECT that has been strongly supported by the Chinese government, and students have learned ECT quickly through various channels.

In short, the role of environmental knowledge in environmental education should not be ignored but should enrich environmental knowledge by adding ECT to the environmental knowledge system and improving the environmental knowledge education curriculum. Although some achievements have been made in this research, there are still some shortcomings. The research data included in this paper are few and not comprehensive. For an impact of ECT on university students’ pro-environmental behavior, different schools are likely to have different characteristics, but this paper only studies a small number of students in two universities. The results are not applicable to every university or every region. Therefore, the next step is to carry out studies in other universities and other regions based on the new KAP theoretical model of this paper to provide more data support for the research results of this paper.

## Data Availability Statement

The original contributions presented in the study are included in the article/supplementary material, further inquiries can be directed to the corresponding author/s.

## Author Contributions

KW conceived the idea for this study. LZ conducted the statistical analysis. Both authors contributed to the final write-up and reviewed and approved the submission.

## Conflict of Interest

The authors declare that the research was conducted in the absence of any commercial or financial relationships that could be construed as a potential conflict of interest.

## Publisher’s Note

All claims expressed in this article are solely those of the authors and do not necessarily represent those of their affiliated organizations, or those of the publisher, the editors and the reviewers. Any product that may be evaluated in this article, or claim that may be made by its manufacturer, is not guaranteed or endorsed by the publisher.
